# Uveitis increases the risk of stroke among patients with ankylosing spondylitis: A nationwide population-based longitudinal study

**DOI:** 10.3389/fimmu.2022.959848

**Published:** 2022-10-06

**Authors:** Ta-hsin Tsung, Ke-Hao Huang, Wu-Chien Chien, Yi-Hao Chen, I-Chuan Yen, Chi-Hsiang Chung, Jiann-Torng Chen, Ching-Long Chen

**Affiliations:** ^1^ Department of Ophthalmology, Tri-Service General Hospital, National Defense Medical Center, Taipei, Taiwan; ^2^ Department of Ophthalmology, Song-Shan Branch of Tri-Service General Hospital, National Defense Medical Center, Taipei, Taiwan; ^3^ Department of Medical Research, Tri-Service General Hospital, National Defense Medical Center, Taipei, Taiwan; ^4^ School of Public Health, National Defense Medical Center, Taipei, Taiwan; ^5^ Graduate Institute of Life Sciences, National Defense Medical Center, Taipei, Taiwan; ^6^ School of Pharmacy, National Defense Medical Center, Taipei, Taiwan; ^7^ Taiwanese Injury Prevention and Safety Promotion Association, Taipei, Taiwan

**Keywords:** uveitis, ankylosing spondylitis, stroke, cerebrovascular disease, epidemiology

## Abstract

Ankylosing spondylitis (AS) is known to increase the risk of stroke. Among patients with AS, uveitis is the most common extra-articular manifestation. However, no previous investigations have discussed the association between uveitis and the risk for developing stroke in patients with AS. This retrospective cohort study aimed to explore the relationship between uveitis and the incidence of stroke in patients with AS by obtaining medical records from January 1, 2000, to December 31, 2015, from the National Health Insurance Research Database, according to the International Classification of Diseases, 9th Revision, Clinical Modification diagnosis codes. The primary outcome was the incidence of stroke. Pearson’s chi-square test and Fisher’s exact test were used to analyze variables. Kaplan–Meier survival curves and univariate and multivariate Cox proportional hazard regression models with and without Fine and Gray’s competing risk model were used to analyze data. Total 828 AS patients with uveitis and 3,312 AS patients without uveitis were identified. During the follow-up period, 137 patients in the uveitis group and 344 in the non-uveitis group developed stroke. Uveitis is a significant risk factor for stroke development in patients with AS (adjusted hazard ratio = 1.846, p < 0.001). Age, diabetes mellitus, hyperlipidemia, hypertension, congestive heart failure, chronic obstructive pulmonary disease, asthma, coronary artery disease, and atrial fibrillation were associated with a higher risk of stroke. After subgroup analysis, both anterior uveitis and posterior segment involvement were found to increase the risk of stroke in patients with AS. Uveitis is associated with an increased risk in both ischemic and hemorrhagic strokes in patients with AS. Therefore, when uveitis is identified, clinicians should pay more attention to the cerebrovascular risk in patients with AS, especially in those with underlying comorbidities.

## Introduction

Ankylosing spondylitis (AS) is a common autoimmune disease that classically affects the axial skeleton, as well as the hips, shoulders, and peripheral joints, causing severe chronic pain and spinal rigidity. The common extra-articular manifestations of AS include uveitis, psoriasis, and inflammatory bowel disease. Immune cells and innate cytokines are crucial for AS pathogenesis (1). Previously, several studies have reported an increased risk of stroke in patients with AS (2–4). Patients with AS have higher levels of several inflammatory biomarkers such as interleukin 6 (IL-6), C-reactive protein (CRP), and tumor necrosis factor alpha (TNF-α) (5–7). These biomarkers are also known to play an important role in stroke as the mediators of the immune system (8–10). Thus, the possible explanations of increased risk of stroke in patients with AS might be associated to the increase of these inflammation related parameters.

Uveitis is known to be the most common extraarticular clinical manifestation of AS. Up to 40% of patients with AS experience unilateral acute anterior uveitis during the disease process (11). Posterior segment inflammation has also been discussed in AS patients with the presentation of diffuse vitritis, papillitis, retinal vasculitis, pars plana exudates, and/or macular edema (12, 13). Previous studies have mentioned that some neurological disorders may cause inflammation of both the central nervous system and the eye, with the manifestation of uveitis (14, 15). Woolfenden et al. reported cases of primary angiitis of the central nervous system presenting as acute ischemic stroke following the onset of uveitis (16). In addition, uveitis is an intraocular inflammatory condition as well as an indicator of systemic inflammation. Previous investigations have showed the increased expression of circulating CRP, IL-6 and TNF-α in the uveitis (17–19). However, no previous articles have demonstrated an association between uveitis and stroke in patients with AS. Hence, according to above findings, this study aimed to investigate the risk of stroke development in AS patients with uveitis by using the National Health Insurance Research Database (NHIRD) of Taiwan.

## Materials and methods

### Research database

The data used in this study were obtained from the National Health Insurance Research Database (NHIRD) of Taiwan. Launched in March 1995, the National Health Insurance (NHI) program contains comprehensive medical records of more than 99% of Taiwan’s population of 23 million, including various forms of outpatient, inpatient, and emergency healthcare services (20). The NHIRD is derived from the NHI beneficiaries claims data and made available to the public in an electronic format for research purposes. Data related to basic parameters, such as sex, age, length of hospital stays, and discharge diagnosis, can be obtained from the NHIRD.

### Study participants

**Figure 1:**
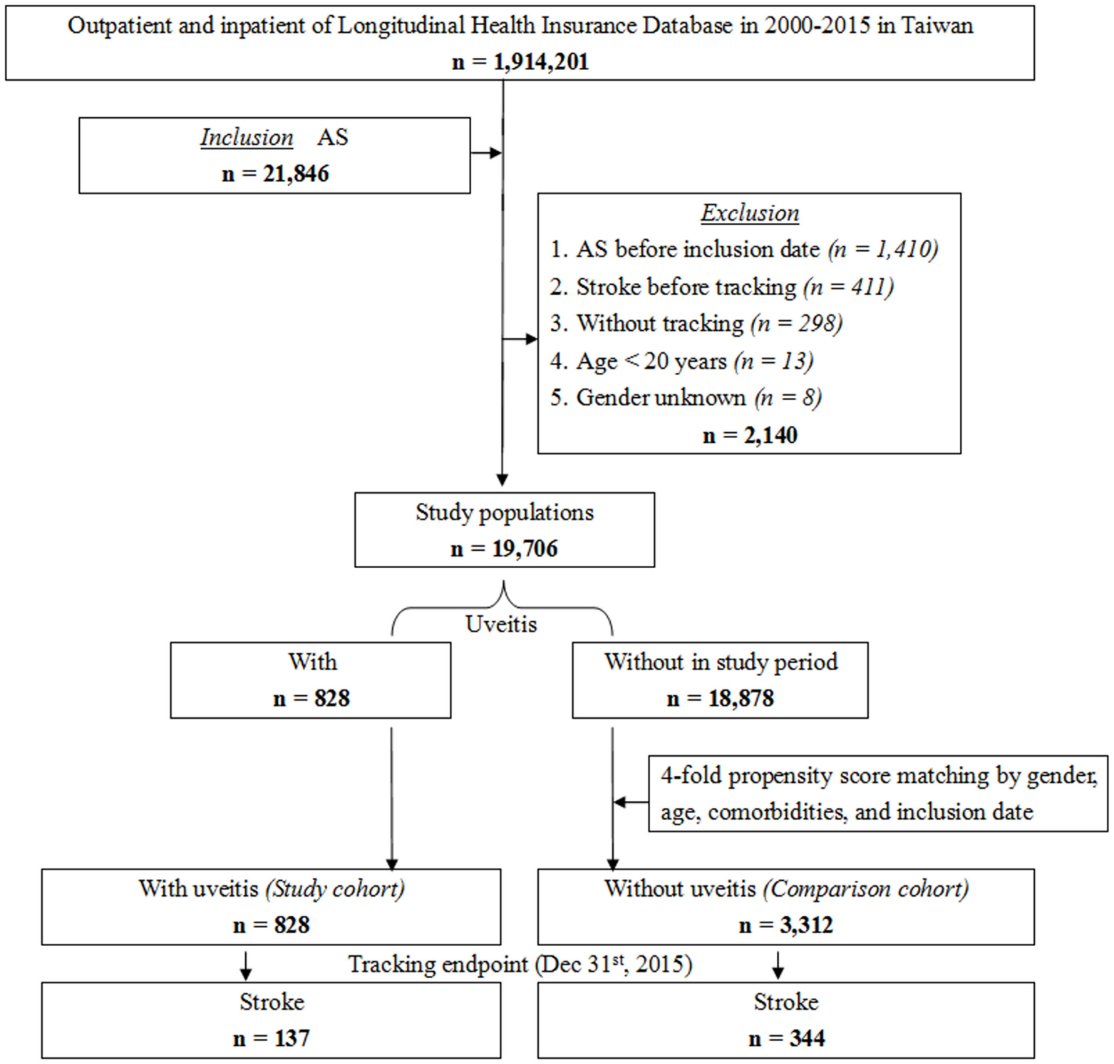
Flowchart of the study.

The NHIRD was used to identify patients diagnosed with AS at least three times during outpatient visits or once during hospitalization according to the International Classification of Disease, 9^th^ Revision, Clinical Modification (ICD-9-CM) code. As shown in **Figure 1**, this longitudinal study included 21,846 patients diagnosed with AS in Taiwan from a total of 1,914,201 patients from January 1, 2000, to December 31, 2015. Among the enrolled patients, 2,140 were excluded. The exclusion criteria were as follows: patients younger than 20 years old (n = 13), AS diagnosed before inclusion date (n = 1,410), stroke diagnosed before the commencement of tracking (n = 411), patients without tracking (n = 298), and patients of unknown sex (n = 8). In total, 19,706 first-time diagnosed AS patients were enrolled in the study during the study period. The study cohort was constructed based on a diagnosis of uveitis among the identified patients with AS. After propensity score matching by sex, age, comorbidities, and inclusion date, the comparison cohort consisted of patients selected from the study population without a diagnosis of uveitis as the non-uveitis group, which was four-fold the number of the uveitis group. All patients were followed up until the incidence of stroke or the tracking endpoint (December 31, 2015).

**Table S1** presents the ICD-9-CM codes and interpretations applied for data extraction and analysis. The comorbidities identified in this cohort study included diabetes mellitus (DM), hyperlipidemia, hypertension (HTN), congestive heart failure (CHF), chronic obstructive pulmonary disease (COPD), asthma, coronary artery disease (CAD), and atrial fibrillation (Af). The Charlson comorbidity index revised (CCI_R) score was recorded with the exclusion of stroke, DM, HTN, CHF, COPD, asthma, and CAD, which were variables in this study. Baseline comorbidities were identified if the patients received medical codes and had at least three medical visits within one year prior to the inclusion date. Comorbidities for endpoints were identified if the patients received medical codes and had at least three medical visits within one year prior to the occurrence of stroke.

### Statistical analysis

All statistical analyses were performed using the SPSS software (version 22.0; IBM Corp., Armonk, NY, USA). Continuous variables are expressed as mean ± SD. Normally distributed continuous data were compared between groups using Student’s t-test. Categorical variables were compared using Pearson’s χ^2^ test. The Kaplan-Meier method was used for the analysis of the cumulative risk of stroke, and the log-rank test was performed to compare the cumulative risk curve of stroke in each year. The Cox proportional hazards regression model was used to estimate the crude and adjusted hazard ratio (HR) for the occurrence of stroke. The assumptions of Cox regression were evaluated by using the Stata software (version 9.0; StataCorp LLC, College Station, TX, USA) with the Schoenfeld residuals test. The result of the global test was not statistically significant (P = 0.8712). Thus, we could assume the Cox regression. The Fine and Gray competing risk regression model was applied for the analysis of time‐to‐event outcomes in the current medical research with the presence of competing events (21). In this study, we used this model to determine the risk of stroke (competing with all-cause mortality) in AS patient with uveitis by presenting as a hazard ratio (HR) with a 95% confidence interval (CI). For conducting the Fine and Gray competing risk regression, we installed the R extension (STATS_COMPRISK.spe version 3.0) for SPSS and then ran the competing risk. Statistical significance was defined as P < 0.05.

## Results

### Characteristics of study patients

**Figure 2:**
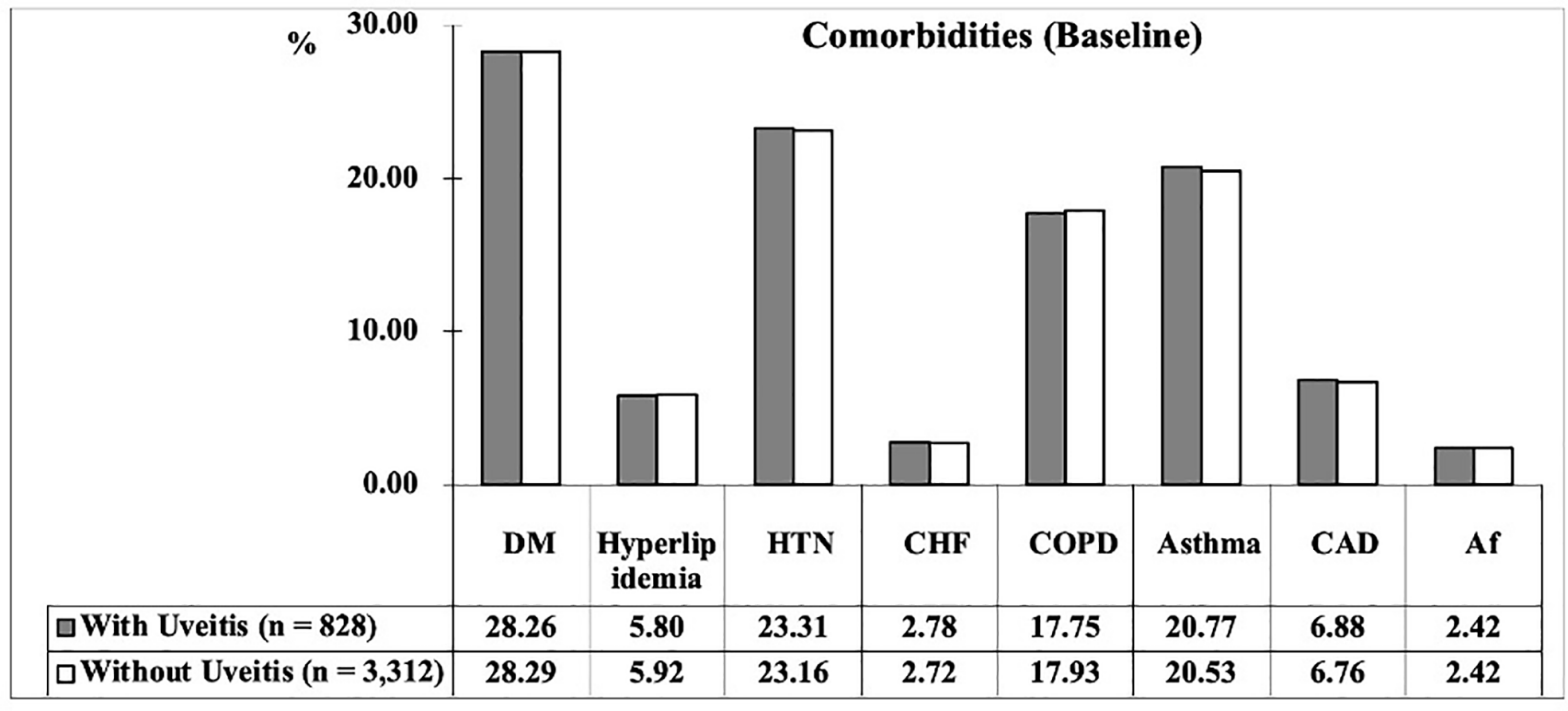
The prevalence of comorbidities between two groups at baseline.

The demographic and baseline characteristics of the AS patients enrolled are presented in **Table 1**. From 2000 to 2015, 4,140 patients with AS were enrolled in total, consisting of 828 patients with uveitis (uveitis group) and 3,312 patients without uveitis (non-uveitis group). The mean age of the AS patients was 37.04 ± 19.21 years. Men and women accounted for 43.12%, and 56.88% of the study population, respectively. The prevalence of DM and HTN in the study population was 28.29% and 23.19%, respectively. Patients with asthma and COPD accounted for 20.58% and 17.90% of the patients, respectively. Fewer patients (< 7%) had hyperlipidemia, CHF, CAD, and Af. At baseline, all the matched listed variables, namely sex, age, comorbidities, and CCI_R score of the two study groups were not statistically significant. The distribution of comorbidities is shown in **Figure 2**.

**Table 1:** Baseline characteristics of the enrolled patients.

Uveitis	Total		With		Without			*P*
Variables	n	%	n	%	n	%		
**Total**	4,140		828	20.00	3,312	80.00		** **
**Gender**								0.999
Male	1,785	43.12	357	43.12	1,428	43.12		
Female	2,355	56.88	471	56.88	1,884	56.88		
**Age (years)**	37.04 ± 19.21		36.95 ± 18.64		37.08 ± 19.80			0.842
**Age group (yrs)**								0.999
20-39	2,415	47.24	483	47.24	1,932	47.24		
40-59	2,125	41.57	425	41.57	1,700	41.57		** **
≧60	572	11.19	114	11.19	458	11.19		** **
**DM**								0.922
Without	2,969	71.71	594	71.74	2,375	71.71		** **
With	1,171	28.29	234	28.26	937	28.29		** **
**Hyperlipidemia**								0.831
Without	3,896	94.11	780	94.20	3,116	94.08		** **
With	244	5.89	48	5.80	196	5.92		** **
**HTN**								0.985
Without	3,180	76.81	635	76.69	2,545	76.84		** **
With	960	23.19	193	23.31	767	23.16		** **
**CHF**								0.703
Without	4,027	97.27	805	97.22	3,222	97.28		** **
With	113	2.73	23	2.78	90	2.72		** **
**COPD**								0.656
Without	3,399	82.10	681	82.25	2,718	82.07		** **
With	741	17.90	147	17.75	594	17.93		** **
**Asthma**								0.843
Without	3,288	79.42	656	79.23	2,632	79.47		** **
With	852	20.58	172	20.77	680	20.53		** **
**CAD**								0.901
Without	3,859	93.21	771	93.12	3,088	93.24		** **
With	281	6.79	57	6.88	224	6.76		** **
**Af**								0.999
Without	4,040	97.58	808	97.58	3,232	97.58		** **
With	100	2.42	20	2.42	80	2.42		** **
**CCI_R**	0.90 ± 1.12		0.93 ± 1.16		0.89 ± 1.11			0.375

DM, diabetes mellitus; HTN, hypertension; CHF, congestive heart failure; COPD, chronic obstructive pulmonary disease; CAD, coronary artery disease; Af, atrial fibrillation; CCI_R, Charlson comorbidity index excluding stroke, DM, hyperlipidemia, HTN, CHF, COPD, asthma, and CAD.

**Figure 3:**
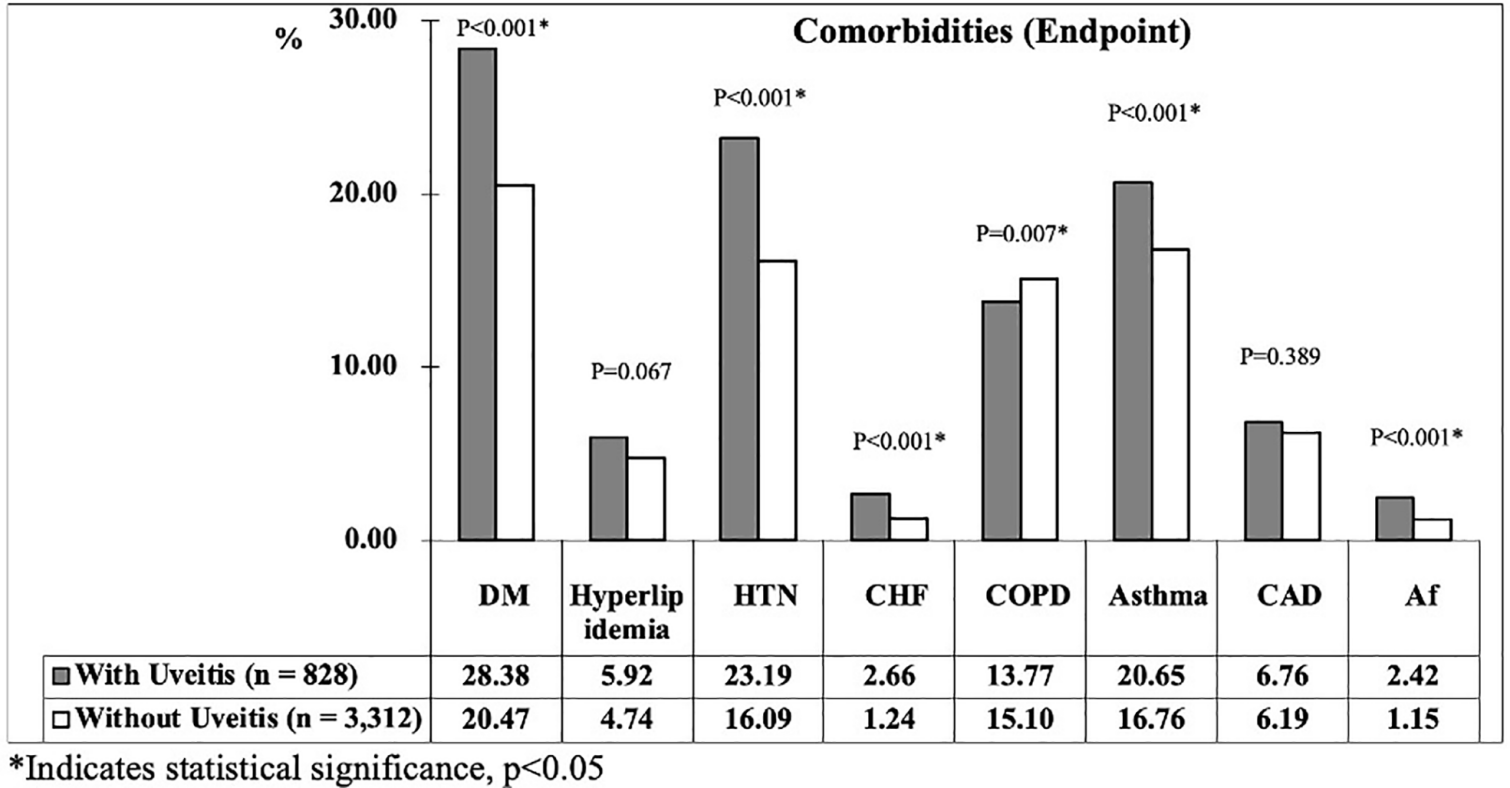
The prevalence of comorbidities between two groups at endpoint.

At the study endpoint, 137 patients (16.55%) in uveitis group and 344 patients (10.39%) in non-uveitis group developed stroke (p < 0.001). The mean age of the uveitis and non-uveitis groups was 41.25 ± 19.79 and 41.55 ± 20.03 years, respectively (p = 0.622). The all-cause mortality, which represents the death rate from all causes of death, was 12.56% and 9.81% in the uveitis and non-uveitis groups, respectively (p = 0.008). The mean follow-up time was 9.58 ± 8.43 (range = 0.01–15.99) years for all patients, and no difference was noted between the uveitis and non-uveitis groups (9.46 ± 8.34 and 9.61 ± 8.44, p=0.502). The mean time of stroke occurrence was 4.24 ± 4.69 years and 4.69 ± 4.73 years (p < 0.001) in the uveitis group and non-uveitis group, respectively. The prevalence of the investigated comorbidities between the two groups at the endpoint is shown in **Figure 3**. The prevalence of the listed comorbidities was significantly higher in the uveitis group than in the non-uveitis group, with the exception of hyperlipidemia and CAD.

### Kaplan-Meier cumulative risk curve for stroke

**Figure 4:**
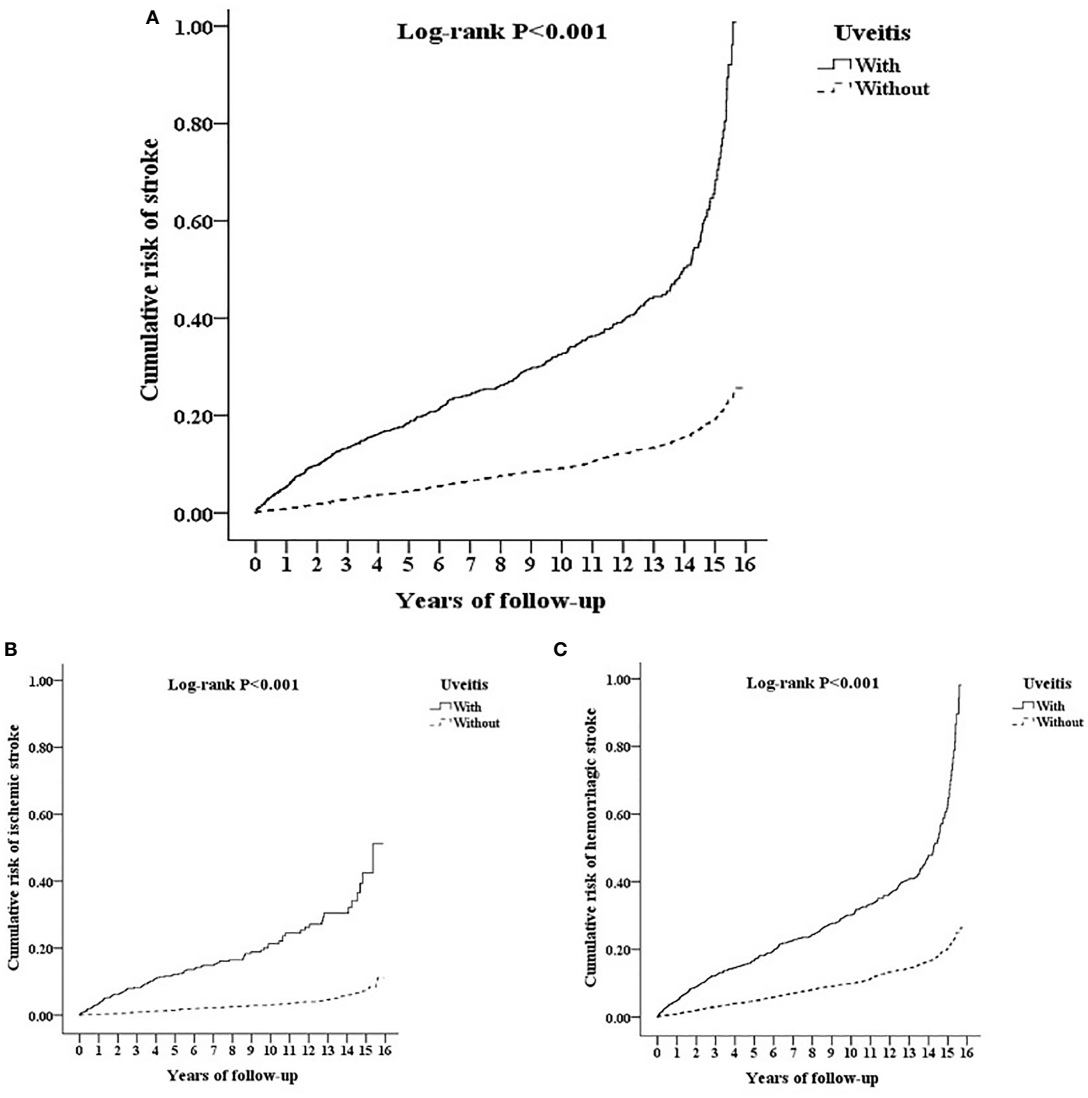
Kaplan-Meier for cumulative risk of **(A)** stroke and subgroups with **(B)** ischemic stroke and **(C)** hemorrhagic stroke aged 20 and over stratified by uveitis with log-rank test.

**Figure 4A** reveals the Kaplan-Meier analysis of the cumulative risk of stroke in AS patients with or without uveitis. AS patients with uveitis had significantly higher cumulative risk of stroke than AS patients without uveitis (p < 0.001; log-rank test). In the subgroup analysis of hemorrhagic and ischemic strokes (**Figures 4B, C**), the uveitis group had a significantly higher risk of developing either type of stroke (p < 0.001) according to the log-rank test.

### Cox regression analysis for risk factors of stroke

**Table 2** shows the risk factors for stroke by Cox proportional hazards regression with and without Fine and Gray’s competing risk model. In the competing risk analysis, the adjusted hazard ratio (aHR) for stroke was 1.846 in the uveitis group (p<0.001). Men were at a higher risk of stroke (aHR = 1.561, p < 0.001). The increased risk of stroke was noted in patients over 40 years old (aHR = 1.515 for the 40–59 age group; aHR = 1.885 for the ≥ 60 age group, all p < 0.001). Stroke risk was significantly higher in AS patients with DM, hyperlipidemia, HTN, CHF, COPD, asthma, CAD, and Af (all p<0.001).

**Table 2:** Risk analysis of stoke by using Cox proportional hazards model with/without Fine & Gray’s competing risk model.

Variables	Without competing risk model				With competing risk model			
	Crude HR (95% CI)	P	Adjusted HR (95% CI)	P	Crude HR(95% CI)	P	Adjusted HR(95% CI)	P
**Uveitis**								
Without	Reference		Reference		Reference		Reference	
With	1.939 (1.775-2.048)	<0.001	1.797 (1.704-1.940)	<0.001	1.987 (1.841-2.078)	<0.001	1.846 (1.749-1.991)	<0.001
**Gender**								
Male	1.555 (1.278-1.798)	<0.001	1.537 (1.258-1.776)	<0.001	1.580 (1.329-1.887)	<0.001	1.561 (1.298-1.879)	<0.001
Female	Reference		Reference		Reference		Reference	
**Age (yrs)**								
20-39	Reference		Reference		Reference		Reference	
40-59	1.512 (1.468-1.644)	<0.001	1.489 (1.441-1.617)	<0.001	1.521 (1.477-1.701)	<0.001	1.515 (1.450-1.668)	<0.001
≧60	1.828 (1.577-1.910)	<0.001	1.768 (1.537-1.894)	<0.001	1.894 (1.590-1.974)	<0.001	1.885 (1.558-1.964)	<0.001
**DM**								
Without	Reference		Reference		Reference		Reference	
With	2.475 (2.053-3.212)	<0.001	2.467 (2.045-3.194)	<0.001	2.553 (2.216-3.333)	<0.001	2.530 (2.117-3.302)	<0.001
**Hyperlipidemia**								
Without	Reference		Reference		Reference		Reference	
With	2.368 (1.796-2.985)	<0.001	2.312 (1.766-2.947)	<0.001	2.471 (1.990-3.212)	<0.001	2.468 (1.938-3.184)	<0.001
**HTN**								
Without	Reference		Reference		Reference		Reference	
With	2.553 (2.068-3.247)	<0.001	2.516 (2.043-3.243)	<0.001	2.664 (2.270-3.458)	<0.001	2.632 (2.179-3.365)	<0.001
**CHF**								
Without	Reference		Reference		Reference		Reference	
With	2.204 (1.779-2.468)	<0.001	2.193 (1.743-2.429)	<0.001	2.247 (1.815-2.483)	<0.001	2.204 (1.780-2.470)	<0.001
**COPD**								
Without	Reference		Reference		Reference		Reference	
With	1.749 (1.476-2.249)	<0.001	1.729 (1.457-2.217)	<0.001	1.791 (1.509-2.316)	<0.001	1.768 (1.485-2.281)	<0.001
**Asthma**								
Without	Reference		Reference		Reference		Reference	
With	1.998 (1.490-2.343)	<0.001	1.991 (1.467-2.320)	<0.001	2.037 (1.537-2.386)	<0.001	2.003 (1.521-2.375)	<0.001
**CAD**								
Without	Reference		Reference		Reference		Reference	
With	2.943 (2.175-3.828)	<0.001	2.907 (2.128-3.809)	<0.001	2.992 (2.227-3.874)	<0.001	2.936 (2.145-3.812)	<0.001
**Af**								
Without	Reference		Reference		Reference		Reference	
With	1.272 (1.130-1.522)	<0.001	1.215 (1.045-1.374)	0.004	1.349 (1.200-1.555)	<0.001	1.222 (1.089-1.414)	<0.001
**CCI_R**	1.581 (1.537-1.592)	<0.001	1.559 (1.530-1.581)	<0.001	1.589 (1.546-1.605)	<0.001	1.577 (1.532-1.584)	<0.001

DM, diabetes mellitus; HTN, hypertension; CHF, congestive heart failure; COPD, chronic obstructive pulmonary disease; CAD, coronary artery disease; Af, atrial fibrillation; CCI_R, Charlson comorbidity index excluding stroke, DM, hyperlipidemia, HTN, CHF, COPD, asthma, and CAD; HR, hazard ratio; CI, confidence interval.

### Factors of stroke stratified in subgroup analysis

The results of stroke risk stratification by the listed variables between AS patients with and without uveitis using Cox proportional hazards regression and Fine and Gray’s competing risk model are listed in **Table 3**. The overall incidence rate for stroke in the uveitis group and non-uveitis group was 1755.44 per 100,000 person-years and 1080.79 per 100,000 person-years, respectively (aHR = 1.846, p < 0.001). Regardless of whether the patients had any stratification variables (sex, age and comorbidities), the aHRs in the uveitis group were significantly higher than those in the non-uveitis group (all p < 0.001 for all stratification). The aHRs were 2.421 and 1.253 in men and women, respectively (p < 0.001). Risk of stroke in patients with uveitis increased regardless of sex (p for interaction = 0.863). Compared with patients without uveitis, patients with uveitis were associated with a higher risk of stroke in all age groups (aHR = 1.763 for the 20-39 age group; aHR = 1.873 for the 40-59 age group; aHR = 3.237 for the ≥ 60 years old, all p < 0.001; p for interaction = 0.365). With respect to comorbidities, the aHRs for stroke in the uveitis group without DM, hyperlipidemia, HTN, CHF, COPD, asthma, CAD, or Af were 1.548, 1.548, 1.502, 1.736, 1.805, 1.736, 1.736, and 1.838, respectively (all p < 0.001). The aHRs for stroke in the uveitis patients combined with DM, hyperlipidemia, HTN, CHF, COPD, asthma, CAD, and Af were 2.752, 2.696, 2.595, 1.870, 2.734, 3.008, 3.896, and 2.334, respectively (all p < 0.001).

**Table 3:** Risk analysis of stroke after stratification by Cox proportional hazards model with/without Fine & Gray’s competing risk model.

Uveitis	With		Without *(Reference)*			Without competing risk model		With competing risk model	
Stratified	Events	Rate (per 10^5^ PYs)	Events		Rate (per 10^5^ PYs)	Adjusted HR (95% CI)	*P*	Adjusted HR (95% CI)	*P*
**Total**	137	1,755.44	344	1,080.79		1.797 (1.704-1.940)	<0.001	1.846 (1.749-1.991)	<0.001
**Gender**									
**Male**	91	2,082.70	174	980.99		2.355 (2.234-2.543)	<0.001	2.421 (2.293-2.609)	<0.001
**Female**	46	1,339.15	170	1,206.41		1.218 (1.156-1.315)	<0.001	1.253 (1.186-1.351)	<0.001
**Age (yrs)**									
**20-39**	86	1,952.04	230	1,272.78		1.714(1.626-1.851	<0.001	1.763 (1.669-1.900)	<0.001
**40-59**	40	1,895.51	99	1,124.33		1.823 (1.728-1.968	<0.001	1.873(1.773-2.019)	<0.001
**≧60**	11	853.75	15	302.87		3.151(2.987-3.401	<0.001	3.237 (3.066-3.491)	<0.001
**DM**									
**Without**	84	1,502.20	278	1,107.74		1.507 (1.429-1.626)	<0.001	1.548 (1.466-1.669)	<0.001
**With**	53	2,395.46	66	980.32		2.678 (2.539-2.891)	<0.001	2.752 (2.606-2.966)	<0.001
**Hyperlipidemia**									
**Without**	95	1,292.98	290	953.67		1.5070 (1.429-1.626)	<0.001	1.548 (1.467-1.669)	<0.001
**With**	42	9,191.18	54	3,803.54		2.623 (2.486-2.832)	<0.001	2.696 (2.533-2.906)	<0.001
**HTN**									
**Without**	82	1,367.33	275	1,031.66		1.460 (1.385-1.577)	<0.001	1.502 (1.422-1.619)	<0.001
**With**	55	3,043.28	69	1,334.00		2.524 (2.394-2.725)	<0.001	2.595 (2.457-2.797)	<0.001
**CHF**									
**Without**	119	1,567.30	322	1,024.84		1.689 (1.601-1.823)	<0.001	1.736 (1.643-1.870)	<0.001
**With**	18	8,504.61	22	5,379.50		1.820 (1.725-1.964)	<0.001	1.870 (1.771-2.016)	<0.001
**COPD**									
**Without**	121	1,890.43	319	1,181.54		1.756 (1.665-1.896)	<0.001	1.805 (1.709-1.946)	<0.001
**With**	16	1,139.87	25	517.61		2.661 (2.523-2.872)	<0.001	2.734 (2.590-2.948)	<0.001
**Asthma**									
**Without**	111	1,791.17	313	1,176.43		1.689 (1.602-1.824)	<0.001	1.736 (1.644-1.871)	<0.001
**With**	26	1,617.67	31	593.57		2.926 (2.775-3.159)	<0.001	3.008 (2.847-3.242)	<0.001
**CAD**									
**Without**	122	1,677.07	329	1,099.40		1.689 (1.602-1.824)	<0.001	1.736 (1.644-1.871)	<0.001
**With**	15	2,831.58	15	788.13		3.791 (3.595-4.092)	<0.001	3.896 (3.690-4.201)	<0.001
**Af**									
**Without**	133	1,746.03	341	1,082.56		1.788 (1.695-1.930)	<0.001	1.838 (1.741-1.982)	<0.001
**With**	4	2,138.69	3	911.72		2.271 (2.154-2.452)	<0.001	2.334 (2.210-2.516)	<0.001

DM, diabetes mellitus; HTN, hypertension; CHF, congestive heart failure; COPD, chronic obstructive pulmonary disease; CAD, coronary artery disease; Af, atrial fibrillation; CCI_R, Charlson comorbidity index excluding stroke, DM, hyperlipidemia, HTN, CHF, COPD, asthma, and CAD; HR, hazard ratio; CI, confidence interval; adjusted HR: adjusted for variables listed in the table; competing risk, All-cause mortality.

To further evaluate the risk of stroke in the different segments of the affected ocular structure in patients with AS, uveitis was classified anatomically into anterior uveitis and posterior segment involvement (**Table 4**). The aHRs in the anterior uveitis and posterior segment involvement groups were 1.844 and 1.900, respectively. The aHR was higher in both subgroups, regardless of the segment involved (p for interaction = 0.862). **Table 5** shows the results of stroke subgroup analysis. The risks were higher in the uveitis group for both hemorrhagic and ischemic stroke (aHR = 1.970 and 1.770, respectively; p < 0.001), but there was no significant difference between the two subgroups (p for interaction = 0.953).

**Table 4:** Risks of stroke among different uveitis subgroups by using Cox proportional hazards model with/without Fine & Gray's competing risk model.

Uveitis subgroup				Without competing risk model		With competing risk model		
	Populations	Events	Rate (per 10^5^ PYs)	Adjusted HR(95% CI)	*P*	Adjusted HR(95% CI)	*P*	*P* for interaction
**Without uveitis**	3,312	344	1,080.79	Reference		Reference		
**With uveitis**	828	137	1,755.44	1.797 (1.704-1.940)	<0.001*	1.846 (1.749-1.991)	<0.001*	
**Anterior uveitis**	353	58	1,755.83	1.765 (1.701-1.925)	<0.001*	1.844 (1.735-1.986)	<0.001*	0.862
**Posterior segment involvement**	475	79	1,755.15	1.846 (1.749-1.990)	<0.001*	1.900 (1.799-2.046)	<0.001*	

*Indicates statistical significance, p<0.05. PYs, Person-years; Adjusted HR, adjusted Hazard ratio: adjusted for the variables listed; CI, confidence interval; Competing risk, All-cause mortality.

**Table 5:** Risks of stroke subgroups by using Cox proportional hazards model with/without Fine & Gray’s competing risk model.

Uveitis	With		Without *(Reference)*		Without competing risk model		With competing risk model		*P* for interaction
Stroke subgroups	Events	Rate (per 10^5^ PYs)	Events	Rate (per 10^5^ PYs)	Adjusted HR(95% CI)	*P*	Adjusted HR(95% CI)	*P*	
**Overall**	137	1,755.44	344	1,080.79	1.797 (1.704-1.940)	<0.001*	1.846 (1.749-1.991)	<0.001*	
**Hemorrhagic stroke**	72	922.56	179	562.39	1.915 (1.819-2.067)	<0.001*	1.970 (1.869-2.168)	<0.001*	0.953
**Ischemic stroke**	65	832.87	165	518.40	1.720 (1.626-1.843)	<0.001*	1.770 (1.666-1.943)	<0.001*	

*Indicates statistical significance, p<0.05. PYs, Person-years; Adjusted HR, adjusted Hazard ratio: adjusted for the variables listed; CI, confidence interval; Competing risk, All-cause mortality.

## Discussion

The major finding of this cohort study was that AS patients with uveitis had a significantly increased risk of stroke occurrence than patients without uveitis, after adjustment for possible confounders and stratified analyses. Based on the Kaplan–Meier analysis, uveitis significantly increased the cumulative risk of stroke in AS patients, irrespective of the hemorrhage or ischemic subgroup. Male sex, age over 40, DM, hyperlipidemia, HTN, CHF, COPD, asthma, CAD, Af, and a higher CCI_R score were significant risk factors for stroke in patients with AS. In stratified analyses, uveitis was significantly associated with an increased risk of stroke, regardless of sex, age, and presence of comorbidities. In the uveitis subgroup analysis, a higher risk of stroke development was presented in both anterior uveitis and posterior segment involvement in patients with AS. The stroke subgroup analysis revealed that uveitis was related to an increased risk of both ischemic and hemorrhagic strokes in patients with AS.

The incidence of uveitis in patients with AS in our study was 4.2% (828/19706 = 4.2%). The occurrence of uveitis in patients with AS varies across studies, ranging from 0% to 40% (22–29). According to previous studies, AS is typically diagnosed in the second to fourth decade of life (30, 31). In our study, the mean age of the total AS patients was 37.04 ± 19.21 years with the majority consistently being 20–39 years old (47.24%). The percentage of AS patients with DM (28.29%) and HTN (23.19%) in our study was higher than those reported in previous studies (32, 33). One Turkish cross-sectional study reported the prevalence of DM and HTN in AS patients to be 13.64% and 20%, respectively (32). Another study in the US reported the percentage of DM among patients with AS to be 17.3% in Caucasians and 27.2% in African Americans (33). The differences in the percentage of comorbidities may have resulted from different races. Owing to matching, baseline characteristics were not statistically different between the uveitis and non-uveitis groups (**Table 1** and **Figure 2**).

The variables related to the stroke development by Cox regression analysis are listed in **Tables 2**,**3**. The risk factors identified in previous studies of stroke development were also noted in our study. Lee et al. found that patients with AS with DM and hyperlipidemia had a higher incidence of ischemic stroke (3). Nisha et al. reported the risk factors for cardiovascular and cerebrovascular death in AS patients to be male sex and age ≥ 65 years (34). A 4-year cohort study that enrolled 8,562 stroke-free people in Taiwan revealed that the incidence rate of first-ever stroke above the age of 35 was 330 per 10^5^ person-years (35). Another population-based stroke survey in Kinmen revealed that the average annual incidence rate of first-ever stroke in people aged over 50 years was 527 per 10^5^ population (36). Compared to previous studies (35, 36), a relatively higher incidence rate of stroke (1,755.44 per 10^5^ person-years in the uveitis group compared with 1,080.79 per 10^5^ person-years in the non-uveitis group) was noted in the present study. The increase of the incidence rate in our study demonstrated that uveitis might play an important role in the stroke development in AS patients.

An NHIRD study revealed that young AS patients (aged 18–45 years) were related to a 1.93-fold increased risk of ischemic stroke (2). Lee et al. reported an increased risk of ischemic stroke (HR=1.46) in patients with AS (3). A 5-year follow-up study in Taiwan reported that male patients with newly diagnosed AS and age greater than 40 years with were at an increased risk of developing cerebrovascular disease (37). Our study also showed that uveitis increased the risk of ischemic stroke (aHR= 1.770).

Although the precise pathogenesis between AS and ischemic stroke remains ambiguous, systemic inflammation may play a major role. Inflammatory alterations are already known to influence stroke susceptibility by leading to blood vessel endothelium dysfunction, coagulation activation, and the initiation or progression of atherosclerosis (38–40). Early features of atherosclerosis have been reported in patients with AS, such as impaired flow-mediated vasodilation and increased carotid intima-media thickness (41–44). The progression of carotid intima-media thickness has been reported as a predictor of stroke in the Multi-Ethnic Study of Atherosclerosis (45). The recruitment of inflammatory cells and the subsequent release of inflammatory cytokines and biomarkers, such as IL-6, TNF-alpha, and CRP, were proposed to be relevant in driving inflammation in AS (5–7). In the meanwhile, these inflammatory parameters were also reported to be elevated in stroke as the mediators of the immune system and the prognostic factors of stroke (8–10). In addition, previous researchers have found the increased expression of circulating CRP, IL-6, and TNF-α in the uveitis (17–19). In this study, we found that uveitis was an independent risk factor for stroke in patients with AS. We speculated that above studies might provide a possible explanation of our findings. However, the detailed mechanism needs further research to identify.

Besides, our results showed that uveitis increased the risk of stroke in AS patient with hypertension. After stroke subgroup analysis (**Table 5**), the p-value for the interaction between the two subgroups showed no statistical significance (p for interaction = 0.953); therefore, the risk exists despite different stroke categories. Berg et al. reported that AS patients with uveitis had an increased odds ratio for hypertension (46). Besides, hypertension is known to be the most common cause of hemorrhagic stroke (47). Long-standing hypertension causes prominent degeneration of the media and smooth muscles of the arteries, leading to subsequent vessel rupture (48). According to previous studies (46–48), hypertension might be one reason that uveitis remained as an independent risk of hemorrhagic stroke in patients with AS.

Our study has several advantages. First, we used data from the NHIRD, which is a validated, comprehensive, longitudinal, and nationwide population-based database with a coverage rate of 99.9% in Taiwan. Second, since 1995, we conducted a 16-year follow-up longitudinal analysis of sequential events based on the NHI system. Patients with AS and stroke diagnosed before the inclusion date were excluded to eliminate bias in our study. The large sample size and long study period provided a good statistical power. Third, matching was conducted between the study groups considering the variables including sex, age, and comorbidities to avoid confounding. The related comorbidities were also adjusted during the statistical analysis for further evaluation. Fourth, we obtained reliable, highly consistent, and convincing results by adjusting for possible confounders in this study using univariate and multivariate Cox proportional hazard regression model and Fine and Gray’s competing risk model. Finally, this study is the first nationwide longitudinal cohort study to indicate that uveitis might be an indepnedent risk factor for the incidence of stroke in patients with AS.

Nevertheless, this study has some limitations. First, given the retrospective cohort design, it is difficult to prove a causal relationship between uveitis and stroke development in patients with AS. Based on the current results, we could only elucidate a possible mechanism. Laboratory examinations such as the level of CRP, erythrocyte sedimentation rate, or other inflammatory cytokines are not recorded in the NHIRD database. Clinical images, such as magnetic resonance imaging findings used to classify AS disease activity and severity, which might correlate with uveitis, or stroke area assessments affecting blood vessels, are also not included in the database. Second, the participants in our study were predominantly Taiwanese; therefore, the effects of different races and ethnicities could not be assessed. Third, we used the ICD-9 coding (Outpatient visits≧ 3 or ≧1 inpatient) as the basis for a diagnosis of uveitis, which have used in several studies (49–51). After literature review, one Hawaii’s study reported that the positive predictive value (PPV) of ICD-9 codes for identifying individual uveitis codes ranged from 0% to 100% and the overall PPV was 61%. In addition, an algorithm that combine ICD codes and medications may help increase the accuracy of diagnosis. Given that the treatment strategy of uveitis is varied, the best method to confirm the diagnosis was the review of medical records. However, there was no report about the accuracy of ICD coding for uveitis in the NHIRD of Taiwan. The accuracy of ICD coding for uveitis diagnosis is one of our limitations. Fourth, another limitation is the inability to account for duration of uveitis exposure from the database. Finally, we used the 2000-2015 NHIRD of Taiwan to analyze the impact of uveitis on the risk of stroke among patients with AS. Considering that the treatment of immune diseases is advanced in recent years, the relationship might change between immune diseases and stroke. Thence, another limitation is that our results might need further verification by new database.

## Conclusion

To summarize, uveitis is an independent risk factor for developing stroke among patients with AS irrespective of the listed clinical variables. When encountering uveitis in patients with AS, clinicians should keep in mind the cerebrovascular risk, especially in those with underlying comorbidities. Careful history taking and closely monitoring the neurological signs and symptoms are crucial in these patients for early detection of stroke, appropriate referral and prompt management for all clinicians. Further prospective studies are still needed to clarify the mechanisms between uveitis and stroke in patients with AS.

## Data availability statement

The original contributions presented in the study are included in the article and **Supplementary Material**. Further inquiries can be directed to the corresponding author.

## Ethics statement

This study was approved by the institutional review board of the Tri-Service General Hospital (TSGHIRB Number: B-110-23) and was performed according to the 1975 Declaration of Helsinki. The identities of all patients included in the database underwent encryption before releasing the data for research purposes; therefore, the requirement for obtaining informed consent was waived by the relevant institutional review board.

## Author contributions

T-HT, W-CC, and C-LC conceived of the presented idea. W-CC, K-HH, and C-HC processed the experimental data. W-CC, I-CY and C-HC performed the data analysis and designed the figures. W-CC, I-CY, and C-HC performed the data acquisition and curation. W-CC, Y-HC, J-TC, and C-LC were involved in planning and supervised the work. T-HT, K-HH, and C-LC aided in interpreting the results and searching literature. T-HT and K-HH wrote the manuscript with support from C-LC. All authors contributed to the article and approved the submitted version.

## Funding

This study was supported by the Medical Affairs Bureau Ministry of National Defense (MAB-108-072, MAB-109-073, MAB-E-109001, and MAB-E-110001) and the Tri-Service General Hospital Research Foundation (TSGH-D-110112 and VTA111-V1-1-2).

## Acknowledgments

This study is based in part on data obtained from the NHIRD, which is provided by the Bureau of National Health Insurance, the Department of Health, Taiwan and managed by the National Health Research Institutes. The authors thank the Tri-Service General Hospital, Taipei, Taiwan, Republic of China, for supporting this study.

## Conflict of interest

The authors declare that the research was conducted in the absence of any commercial or financial relationships that could be construed as a potential conflict of interest.

## Publisher’s note

All claims expressed in this article are solely those of the authors and do not necessarily represent those of their affiliated organizations, or those of the publisher, the editors and the reviewers. Any product that may be evaluated in this article, or claim that may be made by its manufacturer, is not guaranteed or endorsed by the publisher.
