# Supplementary material for: Uveitis increases the risk of stroke among patients with ankylosing spondylitis: A nationwide population-based longitudinal study
Source: Front Immunol. 2022 Oct 6;13:959848. doi: 10.3389/fimmu.2022.959848 (PMC9583155; doi:10.3389/fimmu.2022.959848)
Supplement: Supplementary file 1 [file Table_1.docx]

| Table S1. Abbreviation, ICD-9-CM, and definition | | |
| --- | --- | --- |
|  | **Abbreviation** | **ICD-9-CM / Definition** |
| Study population: |  |  |
| Ankylosing spondylitis | AS | 720.0; Outpatient visits≧ 3 or inpatient |
| Uveitis |  | 363.0, 363.00-363.01, 363.03-363.08, 363.1, 363.10-363.12, 362.18, 363.20-363.21, 364.0-364.3 |
| Anterior uveitis |  | 364.0-364.3 |
| Posterior segment involvement |  | 363.0, 363.00-363.01, 363.03-363.08, 363.1, 363.10-363.12, 362.18, 363.20-363.21 |
| Events: Stroke |  | 430-434, 436 |
| Hemorrhagic stroke |  | 430-432 |
| Ischemic stroke |  | 433-434, 436 |
| Comorbidities: |  | In the baseline: 1 year before inclusion date and medical visits ≧ 3  In the endpoint: 1 year before endpoint and medical visits ≧ 3 |
| Diabetes mellitus | DM | 250 |
| Hyperlipidemia |  | 272 |
| Hypertension | HTN | 401-405 |
| Congestive heart failure | CHF | 428 |
| Chronic obstructive pulmonary disease | COPD | 490-492, 494, 496 |
| Asthma |  | 493 |
| Coronary artery disease | CAD | 410-414 |
| Atrial fibrillation | Af | 427.31 |
| Charlson comorbidity index revised | CCI_R | CCI removed Stroke, DM, HTN, CHF, COPD, Asthma and CAD |

**Table S2. Years to stroke**

|  | | | | | |
| --- | --- | --- | --- | --- | --- |
| **Uveitis** | **Min** | **Median** | **Max** | **Mean ± SD** | ***P*** |
| Total | 0.03 | 4.10 | 15.92 | 4.40 ± 4.72 | <0.001 |
| With | 0.03 | 3.83 | 15.89 | 4.24 ± 4.69 |  |
| Without | 0.03 | 4.20 | 15.92 | 4.69 ± 4.73 |  |
| ***P*: t-test** | | | | | |
